# On delivering polar solvation free energy of proteins from energy minimized structures using a regularized super‐Gaussian Poisson–Boltzmann model

**DOI:** 10.1002/jcc.27496

**Published:** 2024-10-30

**Authors:** Shailesh Kumar Panday, Arghya Chakravorty, Shan Zhao, Emil Alexov

**Affiliations:** ^1^ Department of Physics and Astronomy Clemson University Clemson SC USA; ^2^ Department of Mathematics University of Alabama Tuscaloosa AL USA

**Keywords:** DelPhi, Poisson–Boltzmann equation, polar solvation free energy, protein solvation, regularized PB, super‐Gaussian dielectric

## Abstract

The biomolecules interact with their partners in an aqueous media; thus, their solvation energy is an important thermodynamics quantity. In previous works (*J. Chem. Theory Comput. 14*(2): 1020–1032), we demonstrated that the Poisson–Boltzmann (PB) approach reproduces solvation energy calculated via thermodynamic integration (TI) protocol if the structures of proteins are kept rigid. However, proteins are not rigid bodies and computing their solvation energy must account for their flexibility. Typically, in the framework of PB calculations, this is done by collecting snapshots from molecular dynamics (MD) simulations, computing their solvation energies, and averaging to obtain the ensemble‐averaged solvation energy, which is computationally demanding. To reduce the computational cost, we have proposed Gaussian/super‐Gaussian‐based methods for the dielectric function that use the atomic packing to deliver smooth dielectric function for the entire computational space, the protein and water phase, which allows the ensemble‐averaged solvation energy to be computed from a single structure. One of the technical difficulties associated with the smooth dielectric function presentation with respect to polar solvation energy is the absence of a dielectric border between the protein and water where induced charges should be positioned. This motivated the present work, where we report a super‐Gaussian regularized Poisson–Boltzmann method and use it for computing the polar solvation energy from single energy minimized structures and assess its ability to reproduce the ensemble‐averaged polar solvation on a dataset of 74 high‐resolution monomeric proteins.

## 1 INTRODUCTION

Biomolecules like proteins, DNA, and RNA participate in various cellular processes inside cells whose large volume is occupied by water.^1^ Therefore, the solvation free energy of biomolecules is an essential thermodynamic quantity that influences their stability and interaction with their partners.^2^, ^3^, ^4^, ^5^, ^6^ Thus, determining the solvation free energy of biomolecules is crucial in molecular modeling protocols.^7^ Several methods for modeling the solvation free energy were developed with different computational requirements.^8^, ^9^ Explicit solvent methods like thermodynamic integration (TI), free energy perturbation (FEP), and Bennet's acceptance ratio (BAR) molecular dynamics (MD) simulation are very accurate but computationally expensive.^10^, ^11^ Alternatively, implicit solvent methods like the Poisson–Boltzmann (PB) or Generalized‐Born (GB) can be utilized to compute the polar component of the solvation energy and solvent accessible surface area (SASA) method for accounting for the nonpolar component of the solvation free energy, referred to as PB‐SA or GB‐SA methods.^10^, ^12^, ^13^, ^14^ The implicit solvent methods like PB‐SA or GB‐SA methods offer comparable accuracy at a fractional computational cost compared to the expensive explicit solvent methods like TI. In various application cases like solvation,^10^, ^15^ protein‐ligand complex structure refinement,^16^, ^17^ pKa prediction,^17^, ^18^ folding,^19^ electrostatic component of binding free energy,^12^, ^13^, ^20^ structure prediction,^21^, ^22^, ^23^ constant pH simulation,^24^, ^25^ and predicting the protein‐protein binding free energy^26^ as well as ab‐initio structure modeling of the protein‐ligand,^27^ and protein‐protein^28^ complexes implicit solvent methods like PB‐SA or GB‐GB has been successfully utilized. The above‐mentioned successful applications make the implicit solvent PB/GB approach a popular method of choice. However, if one is concerned with ensemble‐averaged solvation energy, the PB/GB calculations should be done on an ensemble of structures generated via MD simulations, which significantly reduces their speed advantage over FEP/TI. This motivated us to develop the Gaussian‐based smooth dielectric function to enable delivering ensemble‐averaged solvation energy from a single structure.^29^

The PB methods can be grouped into two‐dielectric, multi‐dielectric, and inhomogeneous dielectric methods based on the assignment of dielectric in the modeling space.^30^, ^31^, ^32^, ^33^, ^34^, ^35^, ^36^, ^37^, ^38^, ^39^ In the traditional two‐dielectric method, the space occupied by the solute molecule enclosed by the van der Waals or molecular surface is modeled as a low dielectric region (typically 1 to 20), while the exterior solvent media (usually water) is a high dielectric region (typically 80).^17^ This results in a sharp dielectric border between solute and solvent and offers a molecular surface where induced charges can be positioned.^40^ The approach of induced charges was shown to deliver solvation energy, which is accurate and almost scale‐independent.^38^, ^41^ However, the traditional two‐dielectric model does not account that amino acids have different physico‐chemical properties and thus different dielectric properties as well.^42^, ^43^ To account for the different dielectric properties of individual amino acids, we proposed a multi‐dielectric model where different dielectric constants were assigned to amino acids according to their polarity.^37^ The multi‐dielectric model is physically sound, but it suffers from many sharp dielectric boundaries inside the solute. Additionally, the two‐dielectric and multi‐dielectric methods need an ensemble of structures to compute the solvation energy for every structure, followed by the averaging to deliver the ensemble‐averaged solvation energies.

To address such deficiencies, we proposed an inhomogeneous dielectric model^17^, ^44^ motivated by works on smooth permittivity.^45^ The model is called the Gaussian‐based dielectric method in the framework of the Poisson–Boltzmann equation.^17^, ^46^ It attempts to address two issues: (a) dielectric of the solute regions depends on the packing of individual atoms and amino acids, resulting in high dielectric for loosely packed regions and low dielectric for highly packed, typically hydrophobic regions;^17^ (b) the transition from solute to solvent is not a sharp border as in traditional two‐dielectric or multi‐dielectric methods. Instead, the transition is a smooth dielectric function that reaches solution dielectric as the Gaussian‐based density of solute atoms gets equal to zero.^17^ The method was further extended, and the super‐Gaussian‐based method was proposed.^44^, ^46^ The Gaussian and super‐Gaussian‐based dielectric methods both have been successfully applied in predicting the solvation‐free energy,^29^ the electrostatic component of binding free energy,^47^, ^48^ predicting the p$K_{\text{a}}$ of titratable residues in the proteins,^18^ estimating the entropy of protein‐protein binding^26^ and predicting the localization of mobile ions around the protein surface.^49^ Despite the above‐mentioned successes, the Gaussian‐based methods cannot apply an induced charge approach to make the solvation energy calculations almost grid‐independent as in the traditional two‐dielectric model because of the lack of molecular surface where the induced charges should be positioned. To address this, a regularized PB approach (RPB) was proposed,^50^ and here we report its further development and finite difference (FD) implementation in a Python code based on DelPhi, and its application to deliver ensemble average polar solvation‐free energy for proteins.

In this work, we further develop the super‐Gaussian model, augmenting it with a density‐dependent surface function within the RPB approach over a single energy‐minimized structure and assess its performance by comparing it to the traditional PB model for computing the polar solvation‐free energy over an ensemble of structures for a set of 74 high‐resolution protein structures. The RPB approach allows for computing solvation energy without subtracting grid energies and thus reduces the scale dependence of the modeling.^50^

## 2 THEORY

### 2.1 Poisson–Boltzmann equation for a super‐Gaussian dielectric model

**FIGURE 1:**
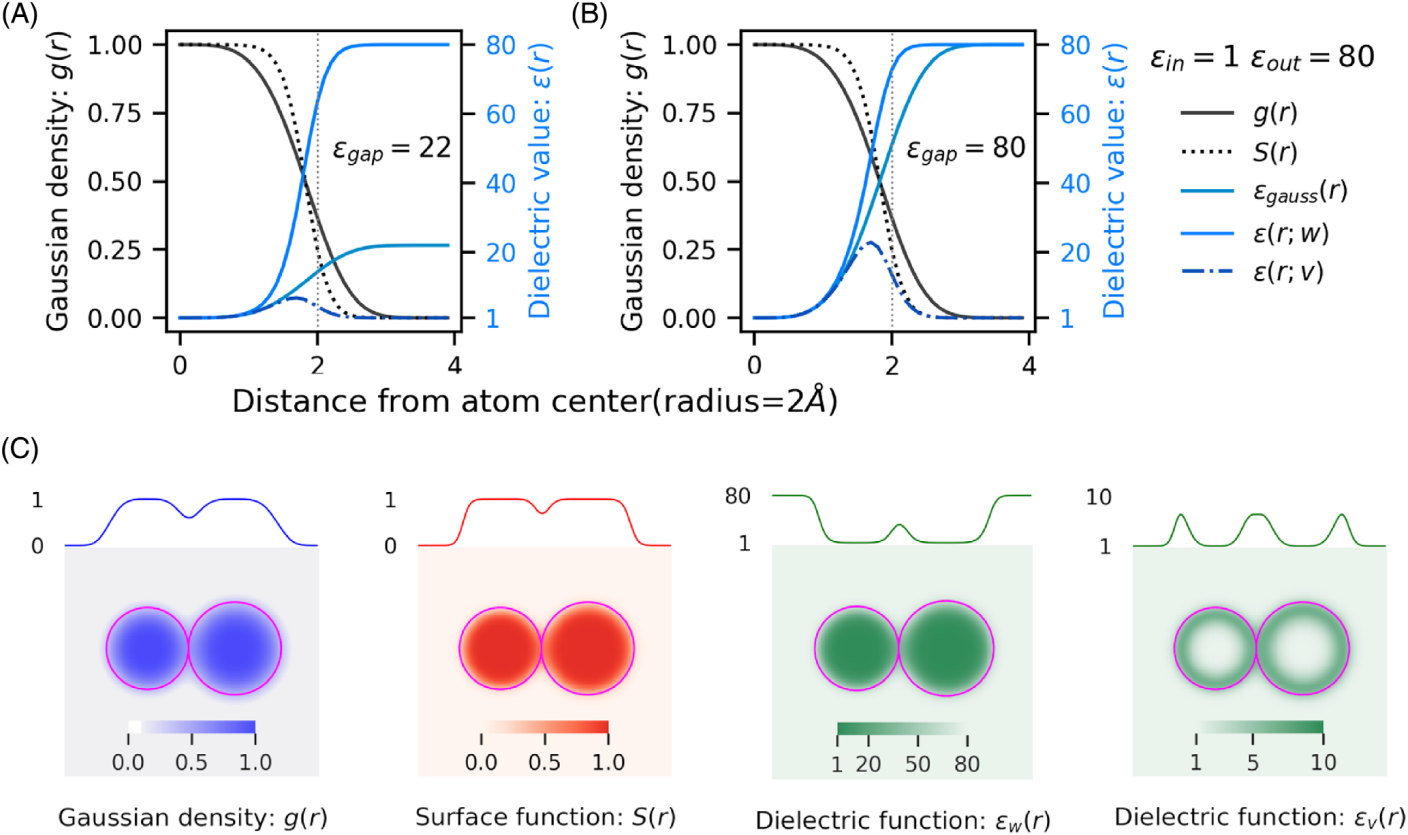
Schematic representation of the density‐dependent surface and dielectric function. The super‐Gaussian density g(r) with m=2, density dependent surface S(r) with η=4/m, and Gaussian density dependent dielectric $\epsilon_{gauss}(r)$ or $\epsilon_{g}(r)$, spatial dielectric in water ϵ(r;w), and spatial dielectric in vacuum ϵ(r;v) are shown for a single atom case where $\epsilon_{ref}=1\;\text{or}\;\epsilon_{in}=1$, and $\epsilon_{\text{out}}=80$, for limiting protein dielectric value: (A) $\epsilon_{\text{gap}}=22$ and (B) $\epsilon_{\text{gap}}=80$. (C) The super‐Gaussian density g(r), density‐dependent surface function S(r), dielectric function in water ϵ(r;w), and dielectric function in vacuum ϵ(r;v) for a two atoms system with radii 1.5, and 1.7 Å positioned at (−1.5, 0, 0) and (1.7, 0, 0) respectively, are shown in XY‐plane passing through z=0 with heatmap, function values at y=0 and z=0 with varying x are shown on top of each subplot, and solid magenta circles show the van der Waals boundary of atoms.

In this section, we describe the super‐Gaussian dielectric model and then detail its use in the Poisson–Boltzmann equation. In the super‐Gaussian dielectric model, the atom is modeled as a density function around its center, which attains a maximum value of one at the center.^17^, ^44^ It decays following a super‐Gaussian‐like function with increasing distance from the center of the atom and becomes zero far away from it (Equation (1)).

(1) $$g_{j}(r)=exp\left(-{\left[\frac{|r-r_{j}{|}^{2}}{\sigma^{2}R_{j}^{2}}\right]}^{m}\right),$$

where $g_{j}(r)$ is the Guassian/super‐Gaussian density due to jth atom at the spatial position r, and $r_{j}$ is the center and $R_{j}$ the radius of the jth atom, respectively. The parameter σ is the variance of the Gaussian distribution, and m is the exponent of the Gaussian distribution; when m=1, then it is termed Gaussian, while m≥2 is termed super‐Gaussian.^44^ A schematic representation showing the influence of variation of parameters Gaussian‐exponent m, and Gaussian variance σ are shown (Supporting Information Figure S1). When m→∞, this model turns into a hard‐sphere model. In the present work, we discuss exclusively the super‐Gaussian model. The total density due to all the $N_{m}$ atoms of the solute at any spatial position r in the modeling space (Ω) is as expressed in Equation (2). An schematic representation of the surface function for a two atom system is shown in Figure 1C. The choice of the value of parameter η is discussed in Section 3.5.

(2) $$g(r)=1-\overset{N_{m}}{\underset{j=1}{\prod }}\left[1-g_{j}(r)\right],$$

The density‐dependent component of the dielectric $\epsilon_{g}(r)$, is given by Equation (3).

(3) $$\epsilon_{g}(r)=\epsilon_{ref}g(r)+\epsilon_{\text{gap}}\left[1-g(r)\right],$$

Using Equation (2), in Equation (3) and simplifying, we obtain a simplified expression for the dielectric function $\epsilon_{g}(r)$ Equation (4).

(4) $$\epsilon_{g}(r)=\epsilon_{ref}+\left(\epsilon_{\text{gap}}-\epsilon_{ref}\right)\overset{N_{m}}{\underset{j=1}{\prod }}\left[1-g_{j}(r)\right],$$

where $\epsilon_{g}(r)$ is the solute density‐dependent component of the dielectric, $\epsilon_{ref}$ is the reference dielectric or minimum dielectric inside the solute region ($\Omega_{i}$), $\epsilon_{\text{gap}}$ is the limiting dielectric inside the solute region. To allow a smooth transition from the solute to the solvent, we also define a Gaussian‐density‐based surface function S(r) (see Equation (5)) as follows:

(5) $$S(r)=\frac{1}{\left[1+{\left(1/g(r)-1\right)}^{\eta }\right],}$$

where η is the parameter that controls the steepness of the density‐dependent surface function S(r). Putting all together, we obtain a smoothed spatial dielectric ϵ(r) at any point r in the modeling space Ω, which is given by Equation (6):

(6) $$\epsilon (r)=S(r)\epsilon_{g}(r)+\left[1-S(r)\right]\epsilon_{\text{out}},$$

where $\epsilon_{\text{out}}$ is the dielectric value of the solvent, the surface function S(r) attains a value of one inside the solute region, and it decays smoothly from one toward zero in the solute‐solvent regions and becomes zero outside. This surface function preserves the dielectric inhomogeneity of the super‐Gaussian dielectric model inside the solute domain and allows a smooth transition to $\epsilon_{\text{out}}$ as point r moves from solute to solvent region. A schematic representation of the (super)‐Gaussian density g(r), density‐dependent surface function S(r), and dielectric functions ϵ(r), for one atom case at two different limiting dielectric values of solute $\epsilon_{\text{gap}}=22$, and $\epsilon_{\text{gap}}=80$ in Figure 1A and B, respectively are shown. A two‐atom case in Figure 1C is shown.

### 2.2 Poisson–Boltzmann equation (PBE)

The nonlinear PBE governing the electrostatic interaction between the solute and solvent in which it is immersed is given by Equation (7):

(7) $$-\nabla .\left(\epsilon (r)\phi (r;u)\right)+\left[1-S(r)\right]\kappa^{2}\sinh(\phi (r;u))=\rho (r)\;\;\;\;\text{in}\;\Omega ,$$

where ϕ(r;u), ϵ(r) and ρ(r) are the potential in solvent phase, dielectric, and source charge density functions, respectively, and $\kappa^{2}$ is salt concentration‐dependent Debye‐Huckle parameter. The expressions for the Debye‐Huckle parameter and the source charge density term due to the partial charges of the atoms of solute are as shown in Equation (8):

(8) $$\kappa^{2}=\left(\frac{2N_{A}e_{c}^{2}}{1000k_{B}T}\right)I,$$

where $N_{A}$ is Avogadro number, $e_{c}$ the charge of the proton in Coulomb unit, $k_{B}$ is the Boltzmann constant, T is the temperature in Kelvin, and I is the salt concentration in mol/liter units. The singular source ρ(r) due to solute atoms (Equation (9)) with respective partial charges $q_{j}$ and center positions $r_{j}$ is as follows:

(9) $$\rho (r)=4\pi \frac{e_{c}^{2}}{k_{B}T}\overset{N_{m}}{\underset{j=1}{\sum }}q_{j}\delta (r-r_{j})\;\;\;\;\;\;\;\;\;\;\;\;\;\;\;\;\;\;\text{in}\;\Omega .$$

Assuming the Ω is big enough so that the dielectric function takes a constant value $\epsilon_{\text{out}}$ at the boundary ∂Ω, then we can assume a Dirichilet‘s boundary condition for the potential in the solvent phase, which at the boundary ∂Ω can be approximated as Equation (10):

(10) $$\phi (r;u)=\phi_{b}(r;u)=\frac{e_{c}^{2}}{k_{B}T}\overset{N_{m}}{\underset{j=1}{\sum }}\frac{q_{j}}{\epsilon_{\text{out}}|r-r_{j}|}e^{-|r-r_{j}|\sqrt{\kappa^{2}/\epsilon_{\text{out}}}}.$$

Alternatively, a computationally efficient dipolar boundary condition, when ∂Ω is considerably far from the partial charges in the solute protein, can be assumed. In this case, the solute's total negative‐ and positive‐partial charges (say $q_{tot}^{-}$ and $q_{tot}^{+}$, respectively) positioned at their respective weighted geometric centers $r_{q_{tot}^{-}}$ and $r_{q_{tot}^{+}}$ form a dipole and give the boundary potential on ∂Ω, as expressed by Equation (11).

(11) $$\begin{array}{ll} \phi_{b}(r;u) & =\frac{e_{c}^{2}}{k_{B}T}\left[\frac{q_{tot}^{+}}{\epsilon_{\text{out}}|r-r_{q_{tot}^{+}}|}e^{-|r-r_{q_{tot}^{+}}|\sqrt{\kappa^{2}/\epsilon_{\text{out}}}}\right.\\ & \;\;\left.-\frac{q_{tot}^{-}}{\epsilon_{\text{out}}|r-r_{q_{tot}^{-}}|}e^{-|r-r_{q_{tot}^{-}}|\sqrt{\kappa^{2}/\epsilon_{\text{out}}}}\right].\; \end{array}$$

where total sum of positive partial charges $q_{tot}^{+}=\sum_{j=1}^{N_{m}}q_{j}\;\;\;\text{if}\;q_{j}>0.0$, is positioned at $r_{q_{tot}^{+}}=(1/q_{tot}^{+})\sum_{j=1}^{N_{m}}q_{j}.r_{j}\;\;\;\text{if}\;q_{j}>0.0$ and total sum of negative partial charges $q_{tot}^{-}=\sum_{j=1}^{N_{m}}q_{j}\;\;\;\text{if}\;q_{j}<0.0$, is positioned at $r_{q_{tot}^{-}}=(1/q_{tot}^{-})\sum_{j=1}^{N_{m}}q_{j}.r_{j}\;\;\;\text{if}\;q_{j}<0.0$. Note that closely positioned $q_{tot}^{+}$ and $q_{tot}^{-}$ would form a dipole of smaller length and tend to have a higher neutralizing effect on the potential due to each other's smaller change in distance from the point of interest and oppositely signed contribution, compared to when they are positioned farther to each other.

### 2.3 Regularization of super‐Gaussian Poisson–Boltzmann equation

To analytically handle the source singularity of the super‐Gaussian Poisson–Boltzmann equation, a dual decomposition of the dielectric and the potential was reported by Wang et al.^50^ In this approach the dielectric is represented as the sum of a constant term $\epsilon_{ref}$, and a position‐dependent variable part $\hat{\epsilon }(r)$ (see Equation (12)), and the potential is represented as a sum of the Coulombs potential term $\phi_{C}(r;u)$ and the reaction‐field component $\phi_{RF}(r;u)$ in a media u (say water) as follows (see Equation (13)):

(12) $$\begin{array}{ll} \epsilon (r) & =\epsilon_{ref}+\hat{\epsilon }(r),\; \end{array}$$

(13) $$\begin{array}{ll} \phi (r;u) & =\phi_{C}(r;u)+\phi_{RF}(r;u),\; \end{array}$$

Assuming the outside medium is water $\epsilon_{\text{out}}=80$ and dielectric at atoms center $\epsilon_{ref}=1$. The variable position‐dependent dielectric part $\hat{\epsilon }(r)$, which is zero in a small neighborhood of the atom's center and $\epsilon_{\text{out}}-\epsilon_{ref}$ in the solvent regions and in between values elsewhere. The Coulomb potential $\phi_{C}(r;u)$ can be assumed to obey a homogeneous Poisson equation with the singular charge source ρ(r) (see Equation (9)) and expressed by Equation (14).

(14) $$\begin{array}{ll} -\epsilon_{ref}\Delta \phi_{C}(r;u) & =\rho (r)\;\;\;\;\;\;\;\text{in}\;{\text{R}}^{3},\\ \phi_{C}(r;u) & =0\;\;\;\;\;\;\;\;\;\;\;\;\text{as}\;r\to \infty .\; \end{array}$$

The singular component $\phi_{C}(r;u)$ is Green‘s function, and it can be given by Equation (15).

(15) $$\phi_{C}(r;u)=G(r)=\frac{e_{c}^{2}}{k_{B}T}\overset{N_{m}}{\underset{j=1}{\sum }}\frac{q_{j}}{\epsilon_{\text{out}}|r-r_{j}|}.$$

After taking out the singular component, the reaction‐field component becomes bounded everywhere, and the PB equation (Equation (7)) can be expanded using the dual decomposition as Equation (16).

(16) $$\begin{array}{ll} & -\nabla .\hat{\epsilon }\nabla \phi_{C}(r)-\nabla .\hat{\epsilon }\nabla \phi_{RF}(r)\\ & -\epsilon_{ref}\nabla \phi_{C}(r)-\epsilon_{ref}\nabla \phi_{RF}(r)\\ & +\left[1-S(r)\right]\kappa^{2}\sinh(\phi_{C}+\phi_{RF})=\rho (r).\; \end{array}$$

Using Equation (13) in 16, singular source term can be eliminated to yield Equation (17):

(17) $$\begin{array}{ll} & -\nabla .\hat{\epsilon }\nabla \phi_{C}(r)-\nabla .\hat{\epsilon }\nabla \phi_{RF}(r)-\epsilon_{ref}\Delta \phi_{RF}(r)\\ & +\left[1-S(r)\right]\kappa^{2}\sinh(\phi_{C}+\phi_{RF})=0.\; \end{array}$$

Upon rearranging the terms, replacing $\phi_{C}(r)$ by known form G(r) (see Equation (15)) and combining $\left[-\nabla .\hat{\epsilon }\nabla \phi_{RF}(r)-\epsilon_{ref}\nabla \phi_{RF}(r)\right]$ into $-\nabla .(\epsilon \nabla \phi_{RF}(r))$ We obtain a regularized PB equation:

(18) $$\begin{array}{ll} & -\nabla .(\epsilon \nabla \phi_{RF}(r))+\left[1-S(r)\right]\kappa^{2}\sinh(\phi_{C}+\phi_{RF})\\ & =\nabla .\left(\hat{\epsilon }(r)\nabla G\right).\; \end{array}$$

In the right‐hand side (r.h.s.) of the regularized PB equation Equation (18), we have a new source term involving the dielectric function $\hat{\epsilon }(r)$ and gradient of the Green's function, for which we have an analytical expression Equation (19):

(19) $$\nabla G(r)=\frac{e_{c}^{2}}{k_{B}T}\overset{N_{m}}{\underset{j=1}{\sum }}\frac{q_{j}}{\epsilon_{\text{out}}|r-r_{j}{|}^{3}}.$$

First, we will discuss the regularity of the new source term $\nabla .\left(\hat{\epsilon }(r)\nabla G\right)$, in the r.h.s. of Equation (18) throughout the domain. The ∇G(r) is smooth everywhere except at the atom's center, where it is singular. Additionally, S(r) is a $C^{2}$ continuous function throughout the domain. Since all the atom centers reside in the domain Ω, so we will examine the regularity of the $\hat{\epsilon }(r)\nabla G(r)$ inside solute region $\Omega_{i}$ only. Inside the $\Omega_{i}$, where S = 1, the variable spatial dielectric is given by Equation (20).

(20) $$\hat{\epsilon }(r)=\left(\epsilon_{\text{gap}}-\epsilon_{ref}\right)\overset{N_{m}}{\underset{j=1}{\prod }}\left[1-g_{j}(r)\right].$$

In a small neighborhood in the vicinity of each atom center $\hat{\epsilon }(r)$ is vanishingly small. Therefore, a rigorous mathematical analysis to examine the regularity was provided by Wang. et. al.^50^ it was shown that $\nabla .(\hat{\epsilon }(r)\nabla G(r))$ in Equation (18), can be replaced by ∇ϵ(r).∇G(r) which is smooth everywhere including at each atom center.

### 2.4 Calculation of electrostatic free energy

In the case of systems where the electrostatic potential is weak, which is common, the nonlinear term sinh(ϕ(r;u)) can be approximated by only the first term of its Taylor series expansion that is, ϕ(r;u) and the electrostatic interactions can be modeled by a linearized PB. In the original super‐Gaussian PB model, the electrostatic energy for the linearized PB equation is given as:

(21) $$-\nabla .\left(\epsilon (r)\nabla \phi (r;u)\right)+\left[1-S(r)\right]\kappa^{2}\phi (r;u)=\rho (r).$$

With the same source term Equation (9), and boundary conditions Equation (10) or Equation (11). Here the ϵ(r) is produced by the super‐Gaussian model, setting $\epsilon_{\text{out}}=80$ for the water phase and solving Equation (21) numerically ϕ(r;u) is obtained. In the case of the vacuum phase, the Poisson equation (Equation (21)), where $\left[1-S(r)\right]\kappa^{2}\phi (r;u)$ in the l.h.s. of Equation (21) vanishes due to the absence of salt ions in the vacuum phase. Setting $\epsilon_{\text{out}}=1$, and using boundary condition (Equation (23)), linearized PB is solved, and the electrostatic potential ϕ(r;v) is obtained.

(22) $$-\nabla .\left(\epsilon (r)\nabla \phi (r;v)\right)=\rho (r),$$

(23) $$\phi_{b}(r;v)=\frac{e_{c}^{2}}{k_{B}T}\overset{N_{m}}{\underset{j=1}{\sum }}\frac{q_{j}}{\epsilon_{\text{out}}|r-r_{j}|}.$$

After calculating the electrostatic potential in the water and the vacuum phase the electrostatic free energy can be calculated by Equation (24).

(24) $$\begin{array}{ll} E & =\frac{k_{B}T}{2}\int_{\Omega }q_{j}\delta (r-r_{j})\left(\phi (r;u)-\phi (r;v)\right)dr\\ & =\frac{k_{B}T}{2}\overset{N_{m}}{\underset{j=1}{\sum }}q_{j}\left(\phi (r_{j};u)-\phi (r_{j};v)\right).\; \end{array}$$

To calculate the electrostatic energy in the regularized Poisson–Boltzmann in the water phase, we plug the value of $\phi (r_{j};u)$ from Equation (13) into Equation (21) and obtain Equation (25):

(25) $$\begin{array}{ll} & -\nabla .\left(\epsilon (r)\nabla \phi_{RF}(r;u)\right)+\left[1-S(r)\right]\kappa^{2}\phi_{RF}(r;u)\\ & =\nabla \epsilon .\nabla G-\left[1-S(r)\right]\kappa^{2}G\;\;\;\text{in}\;\;\Omega ,\;\text{and}\;\\ \phi_{RF}(r) & =\phi_{b}(r;u)-G\;\;\;\;\;\;\;\;\;\;\;\;\;\;\;\;\;\;\;\;\;\text{on}\;\partial \Omega .\; \end{array}$$

In the solute domain $\Omega_{i}$, the $[1-S(r)]\kappa^{2}G(r)=0$, while in the other domain, the Green's function G(r) is well defined. Thus, the second source term of the Equation (25) is well defined over the entire Ω. After solving Equation (25) we obtain the $\phi_{RF}(r)$ for the water phase which is represented as $\phi_{RF}(r;u)$. We can do a similar potential decomposition for the vacuum phase with $\phi (r;v)=\phi_{RF}(r;v)+\phi_{C}(r;v)=\phi_{RF}(r;v)+G(r)$ in Equation (21). Here $\phi_{C}(r;v)$ is essentially the same as $\phi_{C}(r;u)$; this yields a reaction‐field potential satisfying the Poisson–Boltzmann equation (Equation (26)).

(26) $$\begin{array}{ll} -\nabla .\left(\epsilon (r)\nabla \phi_{RF}(r;v)\right) & =\nabla \epsilon_{v}.\nabla G\;\;\;\;\;\;\;\;\;\;\;\;\;\;\;\;\text{in}\;\Omega ,\;\text{and}\;\\ \phi_{RF}(r) & =\phi_{b}(r;v)-G\;\;\;\;\;\;\;\;\;\;\text{on}\;\partial \Omega .\; \end{array}$$

The electrostatic free energy defined by Equation (24) still holds, and the singular components get canceled, leaving only reaction‐field energy, which can be calculated using Equation (27).

(27) $$\begin{array}{ll} E & =\frac{k_{B}T}{2}\int_{\Omega }q_{j}\delta (r-r_{j})\left(\phi (r;u)-\phi (r;v)\right)dr\\ & =\frac{k_{B}T}{2}\overset{N_{m}}{\underset{j=1}{\sum }}q_{j}\left(\phi_{RF}(r_{j};u)-\phi_{RF}(r_{j};v)\right).\; \end{array}$$

Since the numerically calculated reaction field potentials in water and vacuum phase $\phi_{RF}(r;u)$ and $\phi_{RF}(r;v)$, respectively, are known at the grid points, trilinear interpolation is used to find the potential at the charge positions $r_{j}$, and finally the electrostatic free energy is calculated using Equation (27).

## 3 MATERIALS AND METHODS

In this section, we start by describing the dataset of monomeric proteins and outline the process of dataset compilation and protein preparation for energy minimization and molecular dynamics (MD) simulation. Then, we describe the numerical implementation of the RPB and polar solvation energy calculation for energy‐minimized structures and the ensemble of structures generated via MD.

### 3.1 Dataset of representative proteins

**FIGURE 2:**
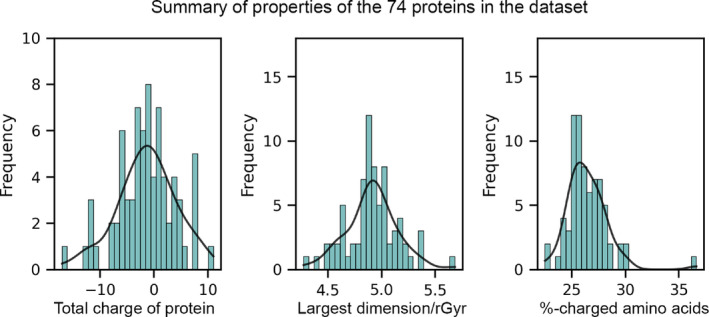
Summary of total charge (left panel), ratio of the largest dimension to radius‐of‐gyration (middle panel), and charged amino acids percentage (right panel) is shown.

We borrowed the dataset compiled by Chakrvorty et al.^29^ containing 74 high‐resolution (0.8 to 0.99Å) proteins. This dataset consisted only of monomeric proteins without any ligands or nonstandard amino acids in the structure. The distribution of net charges is shown in Figure 2A. Among the 74 proteins, the structure with PDB Id 1IQZ has the largest negative charge of −17e, while the PDB Id 1L9L has the largest positive charge of +11e. A total of 68.9% (51 of 74) proteins have a charge between −5e and +5e, while only 9.5% (7 of 74) proteins have an absolute charge greater than 10e. This suggests that the dataset is balanced with respect to net charge of proteins (Figure 2A). Secondly, we analyzed the shape distribution of proteins in the dataset by calculating the distance between the two farthest atoms in the protein, divided it by the radius of gyration of the protein and plotted the frequency plot of this normalized measure in Figure 2B. Thirdly, we analyzed the percentage of the charged amino acids (%ChargedAA) in each protein in the dataset and plotted its frequency. The %ChargedAA varies between 22.5% and 30% for the 73 proteins (Figure 2C). One of the proteins, PDB Id 2PNE, for which it is 36.6%, has a slightly higher %ChargedAA; however, the total charge on the protein is +1e. This suggests that despite having a slightly higher %ChargedAA, this protein is not a distinct member of the dataset.

### 3.2 Protein structure preparation

The structures in the dataset were prepared for MD simulation using GROMACS v5.0.5^51^ using amber ff99SB^52^ force‐field parameters. All the amino acids charged at the physiological pH are kept in the charged state. The system was immersed in a box of TIP3P^53^ water molecules, and counter ions were added whenever needed to neutralize the charge in the solvated system. After the preparation, the system of explicit solvent immersed protein is energy minimized as discussed below; in addition, the protein is also energy minimized in the vacuum and also in Generalized Born implicit solvent^54^ environments.

#### 3.2.1 Energy minimization

The explicit solvated systems were energy minimized for 10,000 steps of steepest descent with harmonic restraint of 1000 to keep the heavy atoms of the protein to their initial positions while allowing others to move freely using GROMACS v5.0.5.^51^ The two other sets of Generalized‐Born implicit solvent (external dielectric 80) and in‐vacuum (external dielectric 1) of minimized structures were also generated. In these two minimizations, 5000 steps of steepest descent with harmonic restraint of 1000 kJ/mol/nm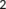 to keep the heavy atoms of the protein to their initial positions while allowing others to freely move using GROMACS v5.0.5 were performed. Here, a smaller number of steps of minimization are used due to the significantly smaller number of atoms in the prepared system compared to the explicit solvent setup.

#### 3.2.2 MD simulation

The energy‐minimized explicit solvent system was equilibrated using a two‐step process, starting with an equilibration for 500 ps in constant volume‐temperature (NVT) condition, followed by 2000 ps, that is, 2 ns equilibration in constant pressure and temperature (NPT) keeping the temperature of the thermal bath to 300 K and the pressure to 1 atmosphere. During the equilibration, the heavy atoms of the protein were harmonically restrained. The equilibrated system configuration is used to start three independent MD simulations using a unique random number seed in each run, in constant pressure‐temperature conditions (NPT ensemble), without any restraint on atoms. During the three production runs, temperature is regulated to 300 K using a velocity scaling scheme, and a constant pressure is maintained using a Parinello–Rehman barostat at a target pressure of 1 atm. The long‐range electrostatic is accounted for using a Particle mesh Ewald scheme with periodic boundary conditions turned on. The coordinates are saved every 10 ps, and each simulation has a total length of 20 ns. The initial 10 ns of each run is discarded to allow for further equilibration upon restraints lifting. This protocol resulted in a total 30 ns MD simulation consisting of 3000 snapshots for each protein in the dataset.

### 3.3 Numerical implementation of RPB

The numerical implementation for solving the regularized PBE, a finite difference method is used. First, we construct a cuboidal mesh around the solute, keeping box dimensions large enough such that potentials as the box boundary can be approximated using the Debye‐Huckle method for the solvent phase and the Coulomb equation for the vacuum. Let's assume that the grid box consists of $N_{x}$, $N_{y}$, and $N_{z}$ grids with the same grid spacing h between two consecutive grids in each of x‐, y‐, and z‐directions that is, h=δx=δy=δz. A function f, defined at grid point $\left(x_{i},y_{j},z_{k}\right)$ that is, $f(x_{i},y_{j},z_{k})$ will be represented as $f_{i,j,k}$ for brevity. Similarly at a grid midpoint between $\left(x_{i},y_{j},z_{k}\right)$ and $\left(x_{i+1},y_{j},z_{k}\right)$ will be represented as $f(x_{i+1/2},y_{j},z_{k})$, $\left(x_{i-1},y_{j},z_{k}\right)$ and $\left(x_{i},y_{j},z_{k}\right)$ with $f(x_{i-1/2},y_{j},z_{k})$, $\left(x_{i},y_{j},z_{k}\right)$ and $\left(x_{i},y_{j+1},z_{k}\right)$ with $f(x_{i},y_{j+1/2},z_{k})$, $\left(x_{i},y_{j-1},z_{k}\right)$ and $\left(x_{i},y_{j},z_{k}\right)$ with $f(x_{i},y_{j-1/2},z_{k})$, $\left(x_{i},y_{j},z_{k}\right)$ and $\left(x_{i},y_{j},z_{k+1}\right)$ with $f(x_{i},y_{j},z_{k+1/2})$, and $\left(x_{i},y_{j},z_{k-1}\right)$ and $\left(x_{i},y_{j},z_{k}\right)$ with $f(x_{i},y_{j},z_{k-1/2})$. The finite difference discretization of the Equation (25) gives rise to a $N_{grids}$‐by‐$N_{grids}$ system of sparse‐linear equation where $N_{grids}=N_{x}\times N_{y}\times N_{z}$. We used a successive over‐relaxation iterative method for solving for $\phi_{RF}(r)$ at each of $N_{grids}$ grid points, detailed elsewhere.^36^

The Green‘s function G(r) and its gradient ∇G(r) of the source term can be calculated at each grid point analytically using Equation (15) and Equation (19), respectively. However, for calculating the term $\left[1-S(r)]\kappa^{2}G(r\right)$ of the source, we first need to calculate the surface function S(r) at each grid point that is, $S_{i,j,k}$. We can define the super‐Gaussian density function at each grid point analytically using Equation (2) and use it in Equation (5) to calculate the surface function. Now we must calculate the ∇ϵ(r) given in Equation (29) at each grid point to get both source terms defined at each grid point.

(28) $$\nabla \epsilon (r)=S(r)\nabla \epsilon_{g}(r)+\nabla S(r)\left[\epsilon_{g}(r)-\epsilon_{\text{out}}\right].$$

To calculate the ∇ϵ(r), we additionally need $\nabla \epsilon_{g}(r)$ and ∇S(r). Since we have analytical expressions Equation (4) and Equation (5) for $\epsilon_{g}(r)$ and S(r) respectively. Here, we derive an analytical expression for the $\nabla \epsilon_{g}(r)$.

(29) $$\begin{array}{ll} \nabla \epsilon_{g}(r) & =-(\epsilon_{\text{gap}}-\epsilon_{ref})\overset{N_{m}}{\underset{j=1}{\sum }}\nabla g_{j}(r)\overset{N_{m}}{\underset{k=1,k\neq j}{\prod }}(1-g_{k}(r))\\ & =-(\epsilon_{\text{gap}}-\epsilon_{ref})\overset{N_{m}}{\underset{j=1}{\sum }}\left[\frac{\left(1-g(r)\right)}{\left(1-g_{j}(r)\right)}\right]\nabla g_{j}(r).\; \end{array}$$

Using the expression for $g_{j}(r)$ from Equation (1) we get the expression for $\nabla g_{j}(r)$ as follows:

(30) $$\begin{array}{ll} \nabla g_{j}(r)= & -\left(\frac{2m}{\sigma^{2m}R_{j}^{2m}}\right)exp\left(-{\left[\frac{|r-r_{j}{|}^{2}}{\sigma^{2}R_{j}^{2}}\right]}^{m}\right)\\ & \;\times \;{\left(|r-r_{j}{|}^{2}\right)}^{m-1}(r-r_{j}).\; \end{array}$$

Now using Equation (29) in Equation (28), we get an expression for the $\nabla \epsilon_{g}(r)$ as follows:

(31) $$\begin{array}{ll} \nabla \epsilon_{g}(r) & =(\epsilon_{\text{gap}}-\epsilon_{ref})\overset{N_{m}}{\underset{j=1}{\sum }}\;\left[\frac{\left(1-g(r)\right)}{\left(1-g_{j}(r)\right)}\left(\frac{2m}{\sigma^{2m}R_{j}^{2m}}\right)\right.\\ & \;\left.\times \;exp\left(-{\left[\frac{|r-r_{j}{|}^{2}}{\sigma^{2}R_{j}^{2}}\right]}^{m}\right){\left(|r-r_{j}{|}^{2}\right)}^{m-1}(r-r_{j})\right].\; \end{array}$$

Similarly, we can derive an analytical expression for the

(32) $$\nabla S(r)=\frac{\partial }{\partial r}\left[\frac{1}{1+{\left(1/g(r)-1\right)}^{\eta }}\right].$$

Which upon simplifying can be written as:

(33) $$\begin{array}{ll} \nabla S(r) & =-\frac{{\left(1/g(r)-1\right)}^{\eta -1}}{{\left[1+{\left(1/g(r)-1\right)}^{\eta }\right]}^{2}}\frac{1}{g{\left(r\right)}^{2}}\frac{\partial \left(1/g(r)-1\right)}{\partial r},\; \end{array}$$

where

(34) $$\begin{array}{ll} \frac{\partial g(r)}{\partial r} & =\overset{N_{m}}{\underset{j=1}{\sum }}\left[\frac{\left(1-g(r)\right)}{\left(1-g_{j}(r)\right)}\left(\frac{2m}{\sigma^{2m}R_{j}^{2m}}\right)\right.\\ & \;\left.\times \;exp\left(-{\left[\frac{|r-r_{j}{|}^{2}}{\sigma^{2}R_{j}^{2}}\right]}^{m}\right){\left(|r-r_{j}{|}^{2}\right)}^{m-1}(r-r_{j})\right].\; \end{array}$$

### 3.4 Ensemble average polar solvation energy calculation

The polar solvation with traditional two‐dielectric PB approach using the DelPhi,^38^ a Poisson–Boltzmann equation (PBE) solver with parameters internal dielectric $\epsilon_{in}=1$, external dielectric $\epsilon_{\text{out}}=80$, the salt concentration of 0 mol/liter, perfil 70%, and grid‐scale 2 (2 grids per angstrom or grid‐spacing of 0.5 Å) were calculated for each of the 3,000 snapshots in the ensemble generated using MD simulations as discussed previously and averaged to result in the ensemble‐averaged polar solvation energy. These ensemble average values are taken from our previous work.^29^ A detailed description of the parameters used in DelPhi calculations and their purpose can be found in the associated tutorial.^39^

### 3.5 Polar solvation energy calculation from energy minimized structures using super‐Gaussian‐based regularized PB approach

The polar solvation energy using a super‐Gaussian based regularized PB approach are calculated with an in‐house implementation of the method in a numba^55^ powered CUDA‐enabled Python code which is based on our popular PBE solver DelPhi.^38^ In order to search for the optimal parameter values that yield the best set of polar solvation energy for the energy minimized structure of the 74‐proteins in the dataset compared to ensemble averages polar solvation energies. The tested combinations of parameter values are as follows. The modeling grid box boundaries are kept at least 15 Å away from any solute atoms, the super‐Gaussian variance parameter (σ) is varied from 0.6 to 1.3 in steps of 0.1, the Gaussian‐exponent (m) is chosen 2 or 3, the limiting dielectric value for the Gaussian‐density dependent component of dielectric function that is, gap‐dielectric ($\epsilon_{\text{gap}}$) is tested from the set of 3, 4, 10, 12, 14, 16, 18, 20, 22, 24, 26, 28, 30, 35, 40, 60, and 80. The density dependent surface function exponent η is tested from 4/m, 6/m, and 8/m. The reference dielectric $\epsilon_{ref}=1$, and external dielectric $\epsilon_{\text{out}}=80$. Additionally, the scale, that is, number of grids per angstrom, varies from 1.5 to 4 in steps of 0.5, which will be used to test and discuss the convergence properties of the proposed method. We tested the performance for above mentioned wide ranges of parameters to obtain an optimal set of parameters that yields best performance. The optimal set of parameters are super‐Gaussian variance parameter σ = 1.0, Gaussian‐exponent (m) = 2, $\epsilon_{ref}=1$, external dielectric $\epsilon_{\text{out}}=80$, $\epsilon_{\text{gap}}=22$, surface function exponent η=4/m, and scale = 2.5. After finding the optimal parameter values, the polar solvation energy results for single energy minimized structures using the optimal parameters are considered for detailed analysis.

## 4 RESULTS AND DISCUSSION

**FIGURE 3:**
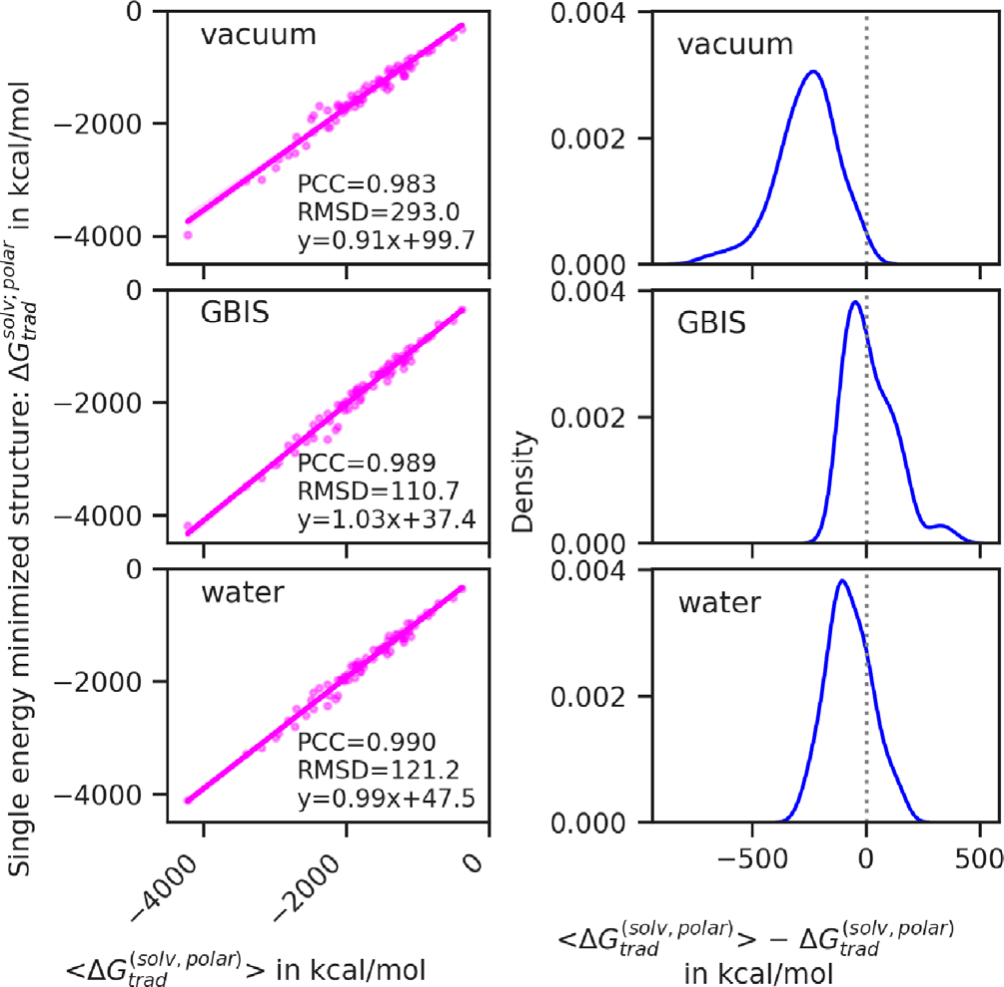
The scatter plot with the fitted regression line and fit‐parameters (column 1) and the density distribution of the difference of ensemble average and that from single energy minimized structure (column 2) are shown for three sets of energy minimized structures in a vacuum, Generalized Born implicit solvent and TIP3P explicit solvent arranged row‐wise from top to bottom.

We start with presenting the results of polar solvation energy using the traditional two‐dielectric PB method. Calculated for energy minimized structures for each of 74 proteins in the dataset in three different environments: vacuum, Generalized‐born implicit solvent and an explicit TIP3P solvent. Afterward, we compare these against the ensemble average polar solvation energy obtained from the same method and set of parameters computed over the ensemble of 3,000 snapshots from three independent simulations for each of the 74 proteins in the dataset. The parameter values used are internal dielectric $\epsilon_{in}=1$, external dielectric $\epsilon_{\text{out}}=80$, salt concentration 0 mol/liter, percent fill 70%, and scale or number of grids per Å 2. These results are summarized in the Figure 3.

In case of a perfect estimation of $\left\langle \Delta G_{trad}^{solv;polar}\right\rangle$ from $\Delta G_{trad,X}^{solv;polar}$ over energy‐minimized structure in environment X a Pearson's correlation coefficient (PCC) of 1, slope of fit 1, intercept of fit 0 kcal/mol and a root mean square deviation (RMSD) very close to zero is expected. The $\Delta G_{trad,X}^{solv;polar}$, and $\left\langle \Delta G_{trad}^{solv;polar}\right\rangle$ both are negative numbers, so when the magnitude of $\Delta G_{trad,X}^{solv;polar}$ is smaller than the magnitude of $\left\langle \Delta G_{trad}^{solv;polar}\right\rangle$, it represents a case where $\Delta G_{trad,X}^{solv;polar}$ is an underestimation of $\left\langle \Delta G_{trad}^{solv;polar}\right\rangle$ from single energy minimized structure and vice versa.

### 4.1 Polar solvation from energy minimized structure underestimates ensemble average calculated with traditional PB

We calculated the polar solvation‐free energy from single energy minimized structure performed in water, Generalized Born implicit solvent (GBIS), and vacuum environments. Irrespective of the energy minimization protocol, there is always a positive intercept of the fit line (first column), suggesting polar solvation energy is underestimated when the single structure is used (Figure 3). The same is evident from the distribution of the energy differences shown in (Figure 3, right column). Setting the internal dielectric to less than 1.0 may be able to mitigate the underestimation. However, it is unphysical to use a smaller dielectric than one.

**FIGURE 4:**
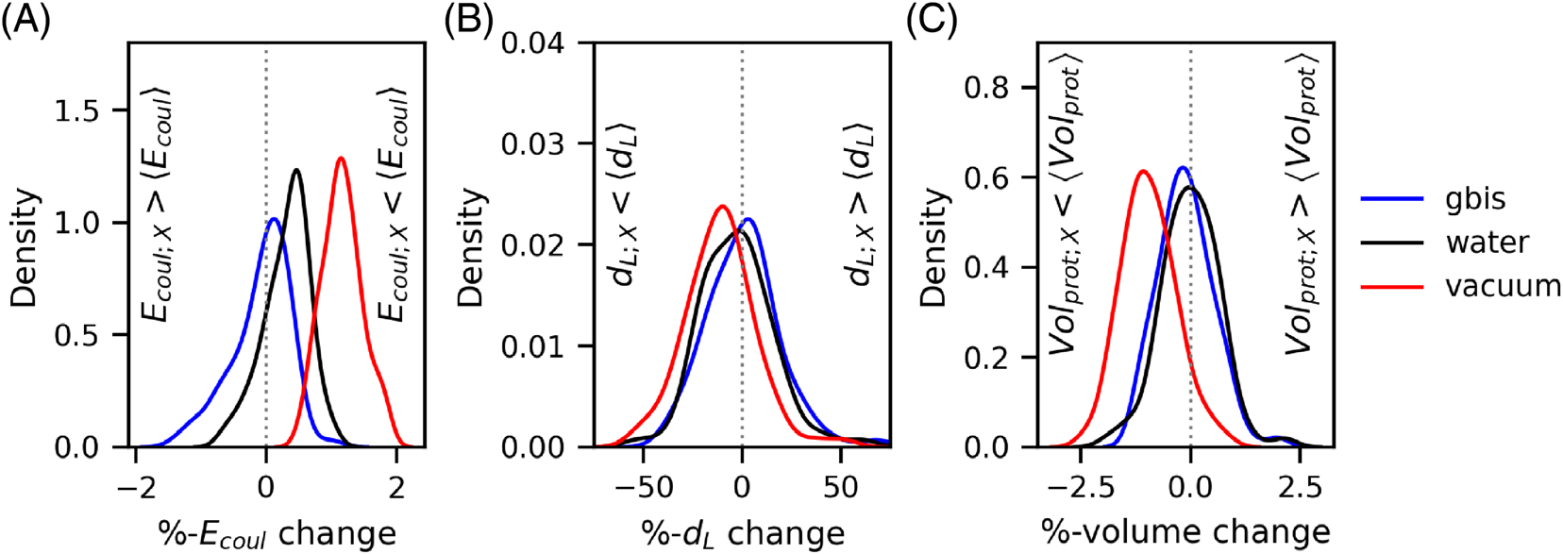
The distribution of the percentage change in (A) coulombs energy, (B) dipole‐length, or (C) volume of the protein calculated over the single energy minimized structure (using protocol X, where X represents GBIS, explicit TIP3P water, or vacuum) with reference to the ensemble average of the same quantity over the snapshots from the MD simulations is shown. The percent change for each of the above quantities Q is calculated as: $\%Q_{X}=100\times (Q_{X}-\langle Q\rangle )/Q_{X}$, and Q is one of the coulombs energy ($E_{coul}$), the dipole‐length ($d_{L}$) of dipole formed by the positive‐ and total negative‐charges centers, or volume of the proteins in the dataset.

To investigate the reason why the traditional two‐dielectric method underestimates ensemble average solvation energy, we calculated total coulombs energy, length of dipole formed in the protein due to positions of positive and negative charge centers, and the volume of the protein. Further, we calculated the percentage change in each of these quantities for each of the 74 proteins in the dataset, and the distribution of these percentages changes for total coulombs energy (Figure 4A), dipole‐length (Figure 4B), and volume (Figure 4C) are shown. A positive percentage change of coulombs energy for energy‐minimized structure relative to ensemble average coulombs energy implies that the coulombs energy, which is negative in sign, has a larger magnitude (peak of the distribution toward the positive %‐$E_{coul}$ change for each minimization case) which is due to closer positions of oppositely charges atoms in minimized structure (Figure 4A). This leads to a smaller dipole length for the dipole formed by the positive‐ and negative‐charge center in the protein‘s energy‐minimized structure, which is observed in Figure 4B (larger area covered by distribution toward negative size). This change is more evident in the case of minimization in a vacuum. Note here a smaller dipole in energy‐minimized structure will have a negative %‐change, as it is always positive, unlike the coulombs energy. Similar trends are observed for the %‐change of volume for energy minimized structure compared to the ensemble average volume (Figure 4C).

To assess the associations of these changes with that in the polar solvation‐free energy, we performed a linear least square fit between %‐changes in coulombs energy ($E_{coul}$), dipole length ($d_{L}$), and volume (calculated using vossvolvox,^56^ a tool implementing a grid‐based algorithm for volume of proteins keeping grid spacing to 0.1 Å) against the changes in polar solvation energy from single energy minimized structure compared to the ensemble average polar solvation energy and summarized the results in Table 1. The strong negative correlation (−0.92, −0.94, −0.69, for water, GBIS, and vacuum) with very high significance (*p*‐value < $1.0\times 10^{-10}$, all the cases) between the %‐change in $E_{coul}$ and %‐change in polar solvation w.r.t. ensemble average suggests, a lower $E_{coul}$ (higher magnitude with negative sign) from energy minimized structure relative to ensemble average, suggest in energy minimized structure oppositely signed partially charged atoms are closely placed and show a relatively larger neutralizing effect on each other thereby lead to elevated polar solvation (smaller magnitude with negative sign). This trend is seen in all cases irrespective of the energy minimization environment used (see Table 1). The same point affirmed from the significant correlation (PCC: 0.40, 0.33, 0.39, *p*‐values < $5.0\times 10^{-3}$, water, GBIS, and vacuum, all the cases) between the %‐changes in dipole‐length ($d_{L}$ is always a positive number) and %‐change in polar solvation w.r.t. ensemble average (see Table 1). Here, the positive correlation shows the same association, that is, closely placed oppositely negative‐ and positive‐charge totals, leading to a higher neutralizing effect and shorter dipole length $d_{L}$. Here again, it increases the polar solvation energy (smaller magnitude and negative sign). In the case of %‐change in volume, the correlation is not significant, suggesting a slight change in volume (−2.5 to 2.5 %), does not cause a significant change in the effective radius of the protein (as the radius is proportional to the cube‐root of the volume) so these changes become even smaller for the radius and thus insignificant influence on the change in polar solvation free energy for the protein in the dataset.

**TABLE 1:** %‐change in polar solvation energy from single structure vs. %‐change in protein structure properties like coulombs energy, dipole length, or volume (all relative to respective ensemble average values)^a^.

			Fitted versus %‐change in $\left\langle \Delta G_{trad}^{solv;polar}\right\rangle$			
EM^b^	Quantity	SL^c^	PCC^d^	Slope	Intercept	*p*‐value
Water	$E_{coul}$	**^e^	−0.92	−15.58	0.4	$5.08\times 10^{-30}$
Water	$d_{L}$	*^f^	0.40	0.13	−4.2	$4.83\times 10^{-4\;}$
Water	Volume	ns^g^	0.01	0.13	−4.5	$9.04\times 10^{-1\;}$
GBIS	$E_{coul}$	**	−0.94	−13.92	−0.4	$1.24\times 10^{-34}$
GBIS	$d_{L}$	*	0.33	0.12	0.5	$3.98\times 10^{-3\;}$
GBIS	Volume	ns	−0.11	−1.18	0.5	$3.46\times 10^{-1\;}$
Vacuum	$E_{coul}$	**	−0.69	−14.66	1.9	$7.34\times 10^{-12}$
Vacuum	$d_{L}$	*	0.39	0.14	−13.7	$6.04\times 10^{-4\;}$
Vacuum	Volume	ns	0.20	2.14	−13.3	$8.11\times 10^{-2\;}$

^a^ The percentage change in polar solvation‐free energy is calculated by subtracting the ensemble average polar solvation from that for energy minimized structure, then normalizing by the ensemble average value and multiplying by 100. Similarly, the percentage change in coulombs energy ($E_{coul}$), dipole length ($d_{L}$) formed by total atomic partial negative and positive charges of protein placed at their respective weighted geometric center, and volume is also calculated. Afterward, the linear least square fit between %‐change in polar solvation energy and %‐change in three structure properties is performed individually, and the results are summarized.

^b^ Energy minimization environment.

^c^ Significance level.

^d^ Pearson Correlation Coefficient.

^e^ Highly significant (*p*‐value < $1.0\times 10^{-10}$).

^f^ Significant ($1.0\times 10^{-10}$ < *p*‐value < $5.0\times 10^{-3}$).

^g^ Not significant (*p*‐value > $5.0\times 10^{-3}$).

This analysis enabled us to gain insight into the physical origins of the underestimation of the polar solvation‐free energy over energy‐minimized structure using a traditional so‐called “two‐dielectric” PB approach. This is primarily due to closer placements of oppositely charged partial charges imparting neutralizing effects on each other and on the protein as a whole.

Next, we analyzed the results obtained from the super‐Gaussian regularized PB (suGaussRPB) approach over the same set of energy‐minimized structures and compared that with ensemble average values. To assess the effectiveness of the suGaussRPB approach in delivering the ensemble‐averaged values from the only energy‐minimized structures. An extensive set of parameter values is explored, and results corresponding to a subset of better‐performing parameter values are summarized in Figure S1.

### 4.2 Parameter optimization for super‐Gaussian RPBE

We observe in Figure S1 that the performance of the method is a function of the three parameters m, $\epsilon_{\text{gap}}$, and σ, excluding one parameter called grid‐scale, which controls the resolution of the finite difference methods. The current optimal results are obtained with a scale of 2.5 grids per Å. A detailed analysis of the influence of scale is presented afterward. With the fixed value for the other two parameters, we observed some general trends for the third. For example, at m=2 or m=3 and σ=1, we see that as the $\epsilon_{\text{gap}}$ increases, the correlation improves and the slope of the fit line decreases and the intercept increases monotonically and changes the sign from negative to positive (Supporting Information: two middle heatmaps in Figure S2). This suggests that when $\epsilon_{\text{gap}}$ is very low, for example, $\epsilon_{\text{gap}}=3$, the magnitude of the overestimation of ensemble‐averaged polar solvation free energy is large. However, with increasing $\epsilon_{\text{gap}}$, it becomes smaller and around 22, it attains a minimum value and thereafter, increasing the $\epsilon_{\text{gap}}$ starts causing underestimation as the slope of the fit line becomes smaller than unity and the intercept changes sign to positive and magnitude keeps increasing instead of the overestimation. Similar trends are observed when we fix m, and $\epsilon_{\text{gap}}$ and vary σ. When m is increased from 2 to 3, the slope of the fit increases, suggesting a further increase in the magnitude of the overestimation.

Turning our attention to the physical implications of variations of these parameters, we find that by increasing the Gaussian exponent m, the flatness of the distribution peak increases (Supporting Information Figure S1), which, as a result, sets lower dielectric to a larger volume of space occupied by the solute. We also know from the Born equation that the polar solvation of a charged system with a lower dielectric has a larger magnitude compared to that from a higher dielectric. The observed trend is in unison with the physical expectations. Similarly, an increase in σ increases the width of the super‐Gaussian distribution and therefore, density is farther spread in the space from the solute, or alternatively, it inflates the volume occupied by the solute, leading to a larger solute region or effective radius which is inversely proportional to the polar solvation, again the general trend of decreasing slope with increasing σ, agrees with physical assumptions. The $\epsilon_{\text{gap}}$ has a twofold effect on the dielectric distribution. First, in the water media, an increasing $\epsilon_{\text{gap}}$ sets higher dielectric in the solute regions, where surface function S(r) is close to 1, and they rapidly change to attain the solvent dielectric as S(r) starts transition from 1 to 0 in the solute‐solvent interface regions (see Figure 1C, second panel). Second, the increasing $\epsilon_{\text{gap}}$ allows setting higher dielectric in solute regions than vacuum dielectric; this causes a lower electrostatic potential in vacuum media (see Figure 1A,B). Since the polar solvation‐free energy is dependent on the difference of electrostatic potential in the two media, water and vacuum. The influence of variation of $\epsilon_{\text{gap}}$ has a subtle influence on the reaction field energy or polar solvation‐free energy.

In this section, we presented and discussed the results of predicting polar solvation‐free energy of proteins using the traditional so‐called “two‐dielectric” PB approach from a single energy minimized structure obtained using three different minimization environments: explicit water, Generalized‐Born implicit solvent, and vacuum. Additionally, we assessed their ability to reproduce the ensemble average polar solvation energy, which was obtained from the ensemble of structures which was generated using MD simulations.

### 4.3 Comparison of super‐Gaussian RPBE and traditional PB for polar solvation energy

Having discussed the traditional PB results from a single structure compared to ensemble average polar solvation using the traditional PB approach. We are presenting the super‐Gaussian regularized PB (suGaussRPB) from a single energy minimized structure in three different minimization environments and comparing it against the ensemble average values of polar solvation.

#### 4.3.1 $△G_{RPB;water}^{solv;polar}$ VERSUS $\left\langle △G_{trad}^{solv;polar}\right\rangle$

**FIGURE 5:**
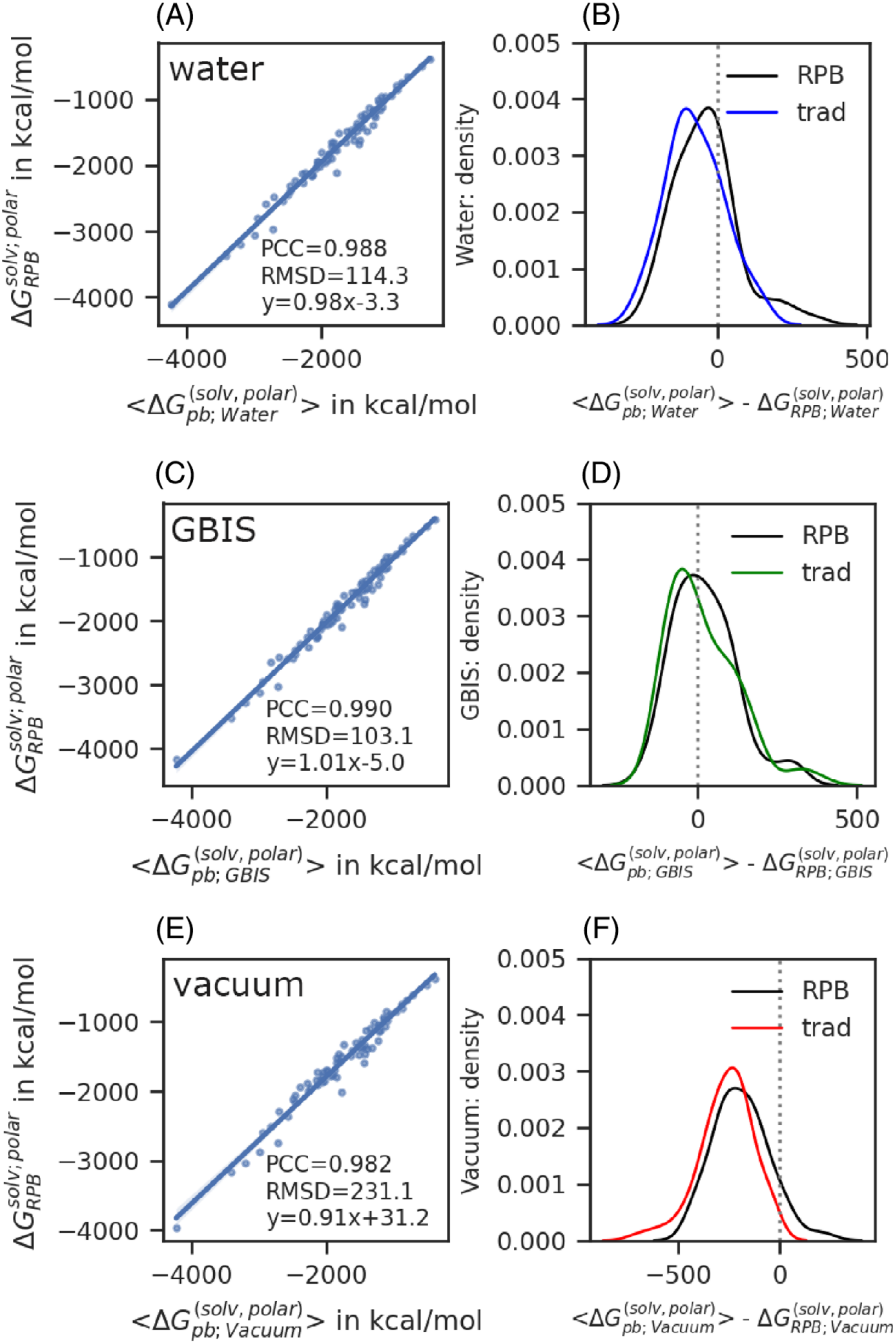
Summary polar solvation from single (A and B) explicit TIP3P water minimized, (C and D) Generalized Born implicit solvent minimized, and (E and F) vacuum minimized set of structures vs ensemble averaged value using the super‐Gaussian regularized PB approach. The parameters used are m=2, σ=1, $\epsilon_{\text{gap}}=22$, $\epsilon_{ref}=1$, $\epsilon_{\text{out}}=1$, with Coulombic boundary condition and scale 2.5 grids per Å. A, C, and E shows the correlation plot for the ensemble‐averaged versus from single energy minimized structure with fit parameters annotated on it. B, D, and F show the distributions of the difference of $\left\langle \Delta G_{trad}^{solv;polar}\right\rangle$ and $\Delta G_{trad,X}^{solv;polar}$ from minimized structure in environment X, where X denotes explicit‐water, GBIS, or vacuum respectively with traditional two‐dielectric (colored curve) or super‐Gaussian RPB (black curve).

Comparing these results to what we obtained from the two‐dielectric method, the correlation is similar to 0.988, while it is 0.99 for traditional 2‐dielectric PB, the slope is also similar to 0.98 with RPB and 0.99 with PB, we notice a significant improvement in the intercept which is −3.3 kcal/mol with RPB and it is was 47.5 kcal/mol with PB. Additionally, we also noticed an improved smaller RMSD of 114.3 kcal/mol compared to 121.3 kcal/mol. Now, the mode of the distribution Figure 5B has moved closer to the zero line, and the distribution has a better balance between the area covered by negative and positive covered regions. This suggests that RPB combined with a newly proposed super‐Gaussian dielectric improves the prediction of ensemble‐averaged polar solvation energy using a single explicit water‐minimized structure. With getting an improved performance over the explicit water minimized structure from suGaussRPB compared to two‐dielectric, we start analyzing the result for GBIS minimized structures.

#### 4.3.2 $△G_{RPB;GBIS}^{solv;polar}$ versus $\left\langle △G_{trad}^{solv;polar}\right\rangle$

The results of polar solvation from a single GBIS minimized structure against ensemble average polar solvation free energy is compiled in Figure S2. Notably, in this case, again, the optimal results correspond to the m=2, σ=1, and $\epsilon_{\text{gap}}=22$. Not only the optimal parameters but also the trend observed in the case of explicit water minimized structures sets holds true here. For example, if we look into the slope and intercept of the fit for parameters m=2, and σ=1.0, and check the influence of increasing $\epsilon_{\text{gap}}$ from 3 to 80. The slope starts at 1.72 and becomes close to 1 at $\epsilon_{\text{gap}}=22$, and becomes 0.72 at 80; at the same time, the intercept starts with −923 kcal/mol and becomes −5 kcal/mol at 22 and changes sign and becomes 352 kcal/mol at 80 (Figure S3, two heatmaps in the middle). With the RMSD being a positive number only, the trend observed in intercept is followed as well, where it starts with a very high RMSD of 2352 kcal/mol, attains a minimum of 103 kcal/mol at 22 and increases again to 896 kcal/mol (Figure S3 right most heatmap). The trend persists with other m and σ values, ascertains the trend is general and changing the other two parameters, the $\epsilon_{\text{gap}}$ may be different, but still, there exists an optimal set of parameters that delivers the ensemble average polar solvation energy.

The $\Delta G_{RPB;X}^{solv;polar}$ vs $\left\langle \Delta G_{trad}^{solv;polar}\right\rangle$ correlation plot and the distribution of differences from the traditional and newly proposed suGaussRPB are shown in Figure 5. As shown in Figure 5C, the RPB gives a slightly better correlation of 0.990 than the traditional 0.989, even closer to one slope of 1.01, which was 1.03 with the traditional two‐dielectric method. Intercept is much smaller, −5.0 kcal/mol compared to 37.4 kcal/mol, and eventually a smaller RMSD, 103.1 kcal/mol compared to 110.7 kcal/mol.

#### 4.3.3 $△G_{RPB;vacuum}^{solv;polar}$ versus $\left\langle △G_{trad}^{solv;polar}\right\rangle$

In the case of a set of structures minimized in a vacuum, we again observe slight improvement with the parameters (m=2, $\epsilon_{\text{gap}}=22$, σ=1.0) that yielded optimal performance in the case of structures energy minimized in GBIS and TIP3P explicit water environments. In this case, we get PCC = 0.982, slope = 0.91, intercept = 31.2 kcal/mol and RMSD = 231.1 kcal/mol. However, the traditional method yielded PCC = 0.983, slope = 0.91, intercept 91.7 kcal/mol, and RMSD 293 kcal/mol. In the case of a set of structures minimized in a vacuum, we again observe slight improvement with the parameters (m=2, $\epsilon_{\text{gap}}=22$, σ=1.0) that yielded optimal performance in the case of structures energy minimized in GBIS and TIP3P explicit water environments. In this case, we get PCC = 0.982, slope = 0.91, intercept = 31.2 kcal/mol and RMSD = 231.1 kcal/mol. However, the traditional method yielded PCC = 0.983, slope = 0.91, intercept 91.7 kcal/mol, and RMSD 293 kcal/mol. The general trends about the influence of increasing $\epsilon_{\text{gap}}$ from 3 to 80 at a fixed m and σ, say m=2, and σ=1.0 still holds. The PCC starts increasing from 0.878, reaches a maximal 0.983, and then decreases to 0.883. The slope starts at 1.57 and reaches 1 and further keeps decreasing to 0.63. The intercept starts with a large magnitude and negative sign −875 kcal/mol, reaches close to zero −3 kcal/mol, and then changes sign, and magnitude increases. Similar trends are seen for RMSD as well, which starts with a very high value of 2023 kcal/mol, decreases to 135 kcal/mol and starts increasing thereafter. It can be argued that even better performance for vacuum‐minimized structures can be obtained at another set of optimal parameters exclusive to vacuum‐minimized structure sets. This makes sense, as due to energy minimization in a vacuum, electrostatic interaction in the interface regions is stronger than explicit or implicit solvent cases due to low dielectric, that is, 1 compared to 80 in solvent cases. This leads to the formation of a higher number of salt bridges as shown by Chakrvorty et al.^29^ Though, one can get a different set of optimal parameters for the set of energy‐minimized structures in a vacuum, performing even better than the common and transferable optimal parameter. We will analyze the results for the common and transferable optimal parameters, which are provided in Figure 5E, F.

As shown in Figure 5E, the PCC is 0.982, the slope is 0.91, the intercept is 31.2 kcal/mol, and RMSD is 231.1 kcal/mol for the vacuum minimized structures using suGaussRPB compared to PCC = 0.983, slope = 0.91, intercept 91.7 kcal/mol, and RMSD 293 kcal/mol from traditional PB. Here, the PCC and slope are similar to the two methods, but intercept and RMSD significantly decrease with suGaussRPB. The point is also clear from the distribution plot of the difference of ensemble‐averaged and from single vacuum minimized structure polar solvation energy (Figure 5F) for the 74 proteins in the dataset. The super‐Gaussian regularized Poisson–Boltzmann (suGaussRPB) outperformed the traditional two‐dielectric PB method in delivering polar solvation‐free energy from single energy minimized structures in a vacuum, GBIS, or explicit TIP3P water environments.

### 4.4 Convergence of suGaussRPB versus Gaussian PB

**FIGURE 6:**
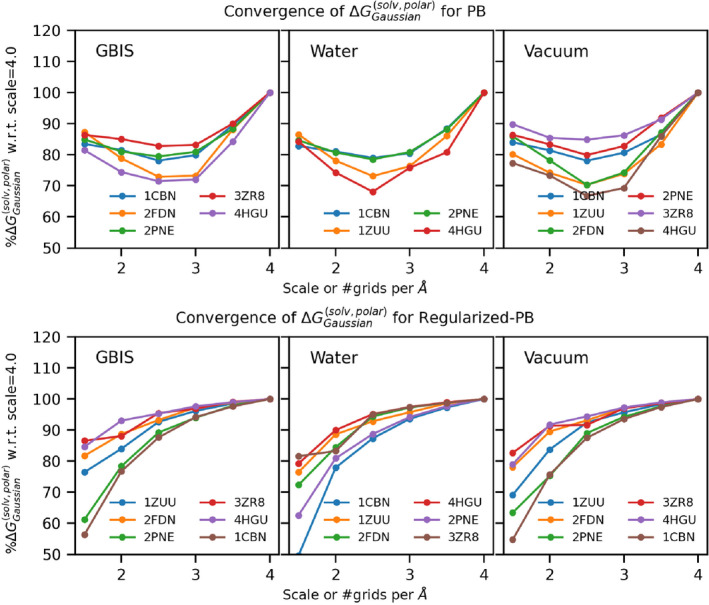
Convergence of polar solvation free energy with respect to scale. The convergence for the selected protein from the dataset for the three sets of energy minimizations is shown on the x‐axis scale used for the calculation, and on the y‐axis, the %‐normalized polar solvation‐free energy with respect to that at scale 4.0 is shown. (A) Gaussian PB (top panel) (B) suGaussRPB (bottom panel).

Here, we assess the convergence properties of the suGaussRPB by comparing it with the convergence of the original Gaussian PB approach for a selected set of proteins. This is done by comparing the normalized polar solvation‐free energy at varying scales from 1.5 to 4.0, incremented in steps of 0.5; the values are normalized to that obtained with scale 4.0. These results are provided in Figure 6. As shown in Figure 6A, the calculated polar solvation energy from the Gaussian PB method makes an upward open concave shape and values at scale 2.0 or 2.5. The values are approximately as low as 70% in some cases. Suggesting a strong dependence on the scale. In contrast with suGaussRPB (Figure 6B), though values are as low as 50% at a low scale of 1.5 with increasing scale, it rapidly approaches 100% in a monotonic manner. At a scale of 2.5 or higher, the values have converged considerably; therefore, all the results discussed in the Results and Discussion section are obtained at a scale of 2.5.

## 5 SUMMARY AND CONCLUSIONS

In the present work, we proposed a regularized Poisson–Boltzmann method and used it in conjunction with the super‐Gaussian density‐dependent dielectric model and a density‐dependent smooth surface function that smoothly separates the solute and the solvent regions of the space. Applied this model to calculate the polar component of the solvation‐free energy for a dataset of 74 proteins. The proteins in the dataset were already resolved with high resolution (0.8 to 0.99Å). The polar component of the solvation‐free energy computed using traditional PB over an ensemble of structures for a set of 19 net‐neutral charged proteins was in excellent agreement (PCC = 0.996, slope of fit line=0.99, intercept of fit −20.22 kcal/mol and RMSD 17.93 kcal/mol)^29^ with that obtained from rigorous thermodynamic integration method^13^ as reported in Chakrvorty et. al.^29^ Previously, it has been shown that Gaussian PB method can reproduce the ensemble‐averaged polar solvation free energy from single energy minimized structure.^29^ However, Gaussian PB is not scale‐independent, unlike traditional PB. The main reason for this is that in Gaussian PB, there is no fixed solute‐solvent boundary where induced charges could be positioned to mitigate scale influences. We developed a super‐Gaussian regularized PB (suGaussRPB) method to address this limitation of the Gaussian PB. The proposed suGaussRPB method is used to deliver the ensemble‐averaged polar solvation energy from a single energy minimized structure, where structures are energy minimized in three different media: vacuum, Generalized‐Born implicit solvent (GBIS) and explicit (TIP3P) water. The ensemble of structures was generated using molecular dynamics simulations, as discussed in the Methods and Materials section. A comparison of the performance of suGaussRPB to traditional PB showed suGaussRPB outperformed it irrespective of whether the energy minimization was done in vacuum, GBIS, or explicit water. Additionally, we tested the convergence of the suGaussRPB against that for Gaussian PB and showed that it converges better than Gaussian PB, that is, shows better scale independence.

## AUTHOR CONTRIBUTIONS

SKP developed the Gaussian‐density‐based surface function and code, carried the calculations and wrote the paper; AC carried MD simulations and calculations with traditional PBE; SZ developed the mathematical formulation of RBPE; and EA supervised the work.

## CONFLICT OF INTEREST STATEMENT

The authors declare no potential conflict of interests.

## Supporting information




**Data S1.** Supporting Information.

## Data Availability

The data that supports the findings of this study are available in the Supporting Information of this article.
