# Supplementary material for: On delivering polar solvation free energy of proteins from energy minimized structures using a regularized super‐Gaussian Poisson–Boltzmann model
Source: J Comput Chem. 2024 Oct 30;46(1):e27496. doi: 10.1002/jcc.27496 (PMC11586710; doi:10.1002/jcc.27496)
Supplement: Supplementary file 1 — Data S1. Supporting Information. [file JCC-46-0-s001.pdf]

Received: Added at production | Revised: Added at production | Accepted: Added at production
DOI: xxx/xxxx

FULL PAPER

On Delivering Polar Solvation Free Energy of Proteins from Energy Minimized Structures using a Regularized super-Gaussian Poisson-Boltzmann Model

Supporting Information:

Shailesh Kumar Panday[1] | Arghya Chakravorty[1] | Shan Zhao[2] | Emil Alexov[1]

[1] Department of Physics and Astronomy, Clemson University, Clemson, South Carolina 29634, USA
[2] Department of Mathematics, University of Alabama, Tuscaloosa, Alabama 35487, USA
Corresponding author Emil Alexov. Email: ealexov@clemson.edu

S1 | NON-SINGULARITY OF DIELECTRIC AND SURFACE FUNCTIONS GRADIENTS

To test the singularity of the dielectric and surface function and their gradients. We start with a one atom model, such that the atoms is placed at $r_1 = (0, 0, 0)$, $\sigma = 1.0$, radius of the atom $R_1 = 1.0$. Any arbitrary point r such that $r = \sqrt{(x^2 + y^2 + z^2)}$.

Then the associated super-Gaussian density

$$g(r) = g_1(r) = e^{-r^4} \tag{1}$$

with Gaussian exponent $m = 2$, then

$$\epsilon_g(r) = \epsilon_{ref} + (\epsilon_{gap} - \epsilon_{ref})\,[1 - g(r)] \tag{2}$$

and

$$\epsilon(r) = S(r)\epsilon_g(r) + [1 - S(r)]\,\epsilon_{out} \tag{3}$$

where

$$S(r) = \frac{1}{1 + (1/g(r) - 1)^2} \qquad \text{with } \eta = 4/m = 4/2 = 2 \tag{4}$$

Using eqn. 1 in eqn. 4, we get

$$S(r) = \frac{1}{1 + [(1 - g(r))/g(r)]^2} = \frac{1}{1 + 1/g(r)^2 + g(r)^2/g(r)^2 - 2g(r)/g(r)^2} = \frac{1}{e^{2r^4} - 2e^{r^4} + 2} \tag{5}$$

$$1 - S(r) = \frac{e^{2r^4} - 2e^{r^4} + 1}{e^{2r^4} - 2e^4 + 2} \tag{6}$$

Now, we shall check if $\hat{\epsilon}(r)\nabla G(r)$ and $\nabla\epsilon(r).\nabla G(r)$ are singular? As $r \to 0$, here

$$\begin{aligned}\hat{\epsilon}(r)\nabla G(r) &= \hat{\epsilon}(r)\frac{dG(r)}{dr}\\ \nabla\epsilon(r).\nabla G(r) &= \frac{d\epsilon(r)}{dr}\frac{dG(r)}{dr}\end{aligned} \tag{7}$$

 © 2024 Copyright Holder Name

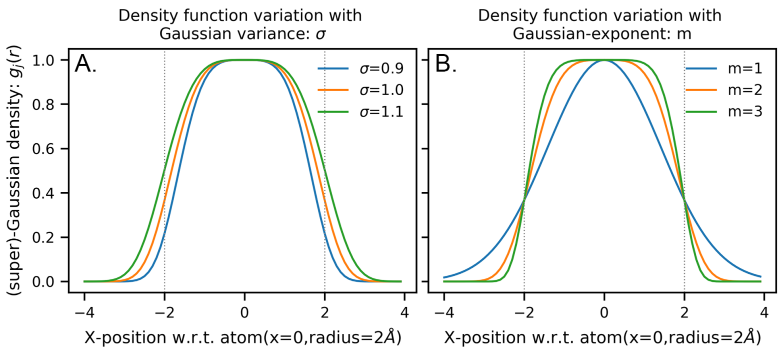

FIGURE S1 The change in the steepness of the Gaussian/super-Gaussian density function for a one-atom system as function of displacement from its center is shown in the left panel. The influence of the parameter σ on the width of the super-Gaussian density function with exponent $m = 2$ is shown in the right panel.

and

$$\frac{dG(r)}{dr} = -\frac{e_c^2}{k_B T}.\frac{q}{\epsilon_{ref}}.\frac{1}{r^2} = C\frac{1}{r^2} \qquad \text{where } C \text{ is a constant} \tag{8}$$

Now taking

$$\lim_{r \to 0} \hat{\epsilon}(r)\frac{dG(r)}{dr} \tag{9}$$

we get

$$\lim_{r \to 0} \frac{\frac{C}{e^{2r^4}-2e^4+2}\left[\epsilon_{ref} + (\epsilon_{gap} - \epsilon_{ref})(1 - e^{-r^4})\right] - C\epsilon_{ref} + C\epsilon_{out}\frac{e^{2r^4}-2e^{r^4}+1}{e^{2r^4}-2e^{r^4}+2}}{r^2} \tag{10}$$

as $r \to 0$, $S(r) \to 1$, $(1 - S(r)) \to 0$, $(1 - e^{r^4}) \to 0$, limit in eqn. 10 $\to \frac{0}{0}$

$$\lim_{r \to 0} \frac{C\left[\epsilon_{ref} + (\epsilon_{gap} - \epsilon_{ref})(1 - e^{-r^4})\right] - C\epsilon_{ref}(e^{2r^4} - 2e^{r^4} + 2) + C\epsilon_{out}(e^{2r^4} - 2e^{r^4} + 1)}{(e^{2r^4} - 2e^{r^4} + 2)r^2} \tag{11}$$

Using L'Hospital's rule.

$$\lim_{r \to 0} \frac{C\left[(\epsilon_{gap} - \epsilon_{ref})4r^3(e^{-r^4})\right] - C\epsilon_{ref}(8r^3e^{2r^4} - 8r^3e^{r^4}) + C\epsilon_{out}(8r^3e^{2r^4} - 8r^3e^{r^4})}{(8r^3e^{2r^4} - 8r^3e^{r^4})r^2 + 2r(e^{2r^4} - 2e^{r^4} + 2)} \tag{12}$$

plugging $r \to 0$ $\hat{\epsilon}\frac{dG}{dr} \to 0$ is non-singular.

Now, lets test the singularity of the $\frac{d\epsilon(r)}{dr}$

$$\frac{d\epsilon(r)}{dr} = \epsilon_g(r)\frac{dS(r)}{dr} + S(r)\frac{d\epsilon_g(r)}{dr} - \frac{dS(r)}{dr}\epsilon_{out} \tag{13}$$

$$\frac{dS(r)}{dr} = -\frac{8r^3(e^{2r^4} - e^{r^4})}{(e^{2r^4} - 2e^{r^4} + 2)^2}$$
$$\frac{d\epsilon_g(r)}{dr} = (\epsilon_{gap} - \epsilon_{ref})4r^3e^{-r^4}$$

$$\begin{aligned}\lim_{r \to 0} \frac{d\epsilon(r)}{dr}\frac{dG(r)}{dr} &= \lim_{r \to 0} \frac{C\left[\epsilon_{out} - (\epsilon_{ref} + (\epsilon_{gap} - \epsilon_{ref})(1 - e^{-r^4}))\right]\frac{8r^3(e^{2r^4}-e^{r^4})}{(e^{2r^4}-2e^{r^4}+2)^2} + \frac{C4r^3e^{r^4}(\epsilon_{gap}-\epsilon_{ref})}{(e^{2r^4}-2e^{r^4}+2)}}{r^2} \\ &= \lim_{r \to 0} \frac{C\left[\epsilon_{out} - (\epsilon_{ref} + (\epsilon_{gap} - \epsilon_{ref})(1 - e^{-r^4}))\right]8r^3(e^{2r^4} - e^{r^4}) + C4r^3e^{r^4}(\epsilon_{gap} - \epsilon_{ref})(e^{2r^4} - 2e^{r^4} + 2)}{r^2(e^{2r^4} - 2e^{r^4} + 2)^2} \\ &= 0\end{aligned} \tag{14}$$

Therefore, The RPBE is well-defined for super-Gaussian density.

S2 | SUPPLEMENTARY FIGURE

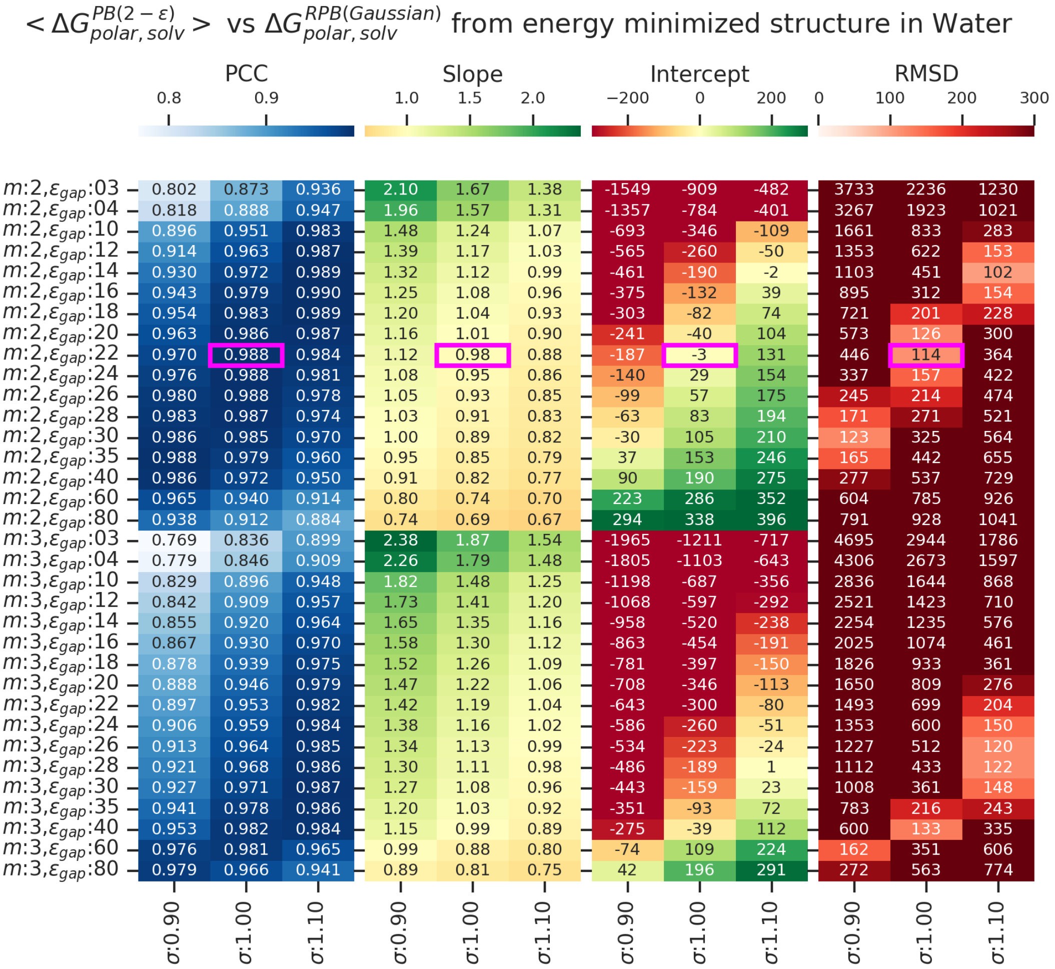

FIGURE S2 Summary of $\Delta G^{solv;polar}_{RPB}$ obtained from single energy minimized structures in explicit solvent using a super-Gaussian regularized PB method. The four performance indicators PCC, slope, intercept (in kcal/mol) and the RMSD (in kcal/mol) are shown as heatmap for a subset (showing only for better performing) of parameters explored. The optimal parameters we found are Gaussian exponent $m = 2$, limiting solute dielectric $\epsilon_{gap} = 22$, and variance of the super-Gaussian distribution $\sigma = 1.0$.

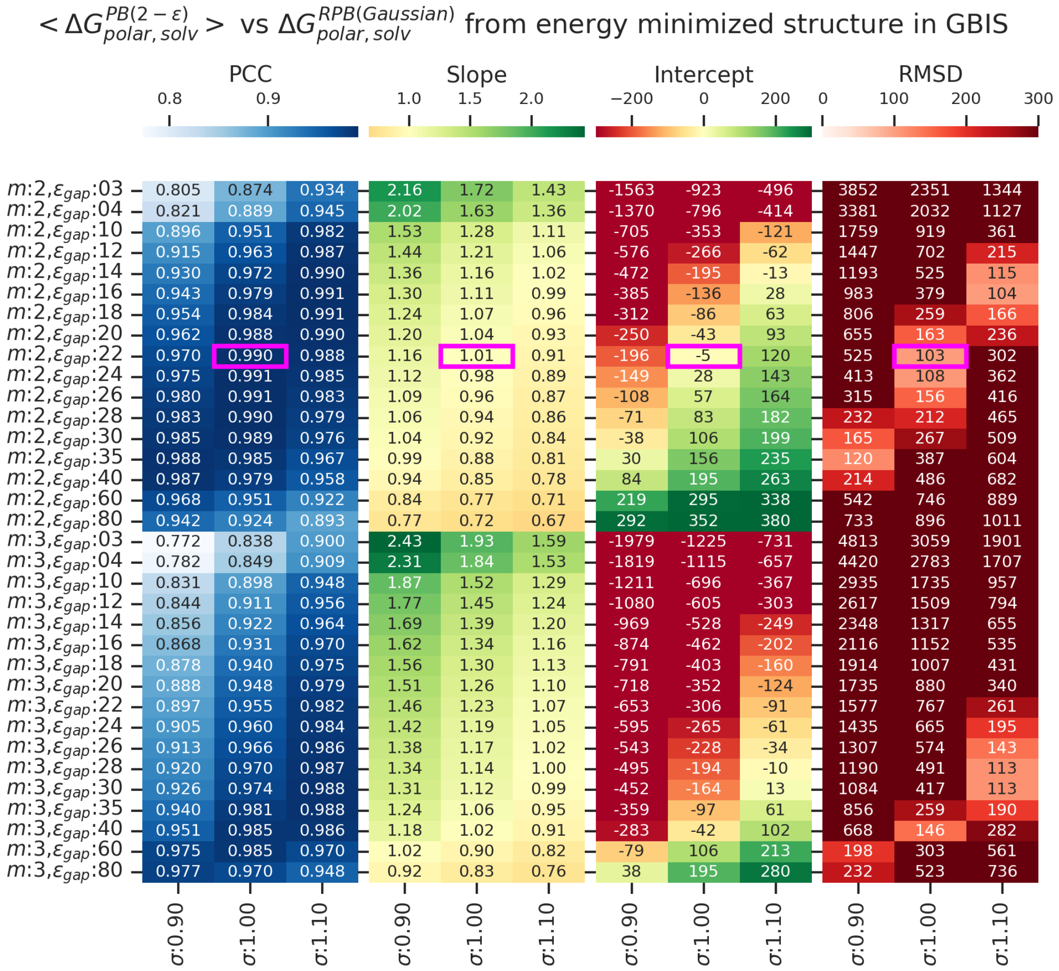

FIGURE S3 Summary of $\Delta G^{solv;polar}_{RPB}$ obtained from single energy minimized structures in GBIS using a super-Gaussian regularized PB method. The four performance indicators PCC, slope, intercept (in kcal/mol) and the RMSD (in kcal/mol) are shown as heatmap for a subset (showing only for better performing) of parameters explored. The optimal parameters we found are Gaussian exponent $m = 2$, limiting solute dielectric $\epsilon_{gap} = 22$, and variance of the super-Gaussian distribution $\sigma = 1.0$.

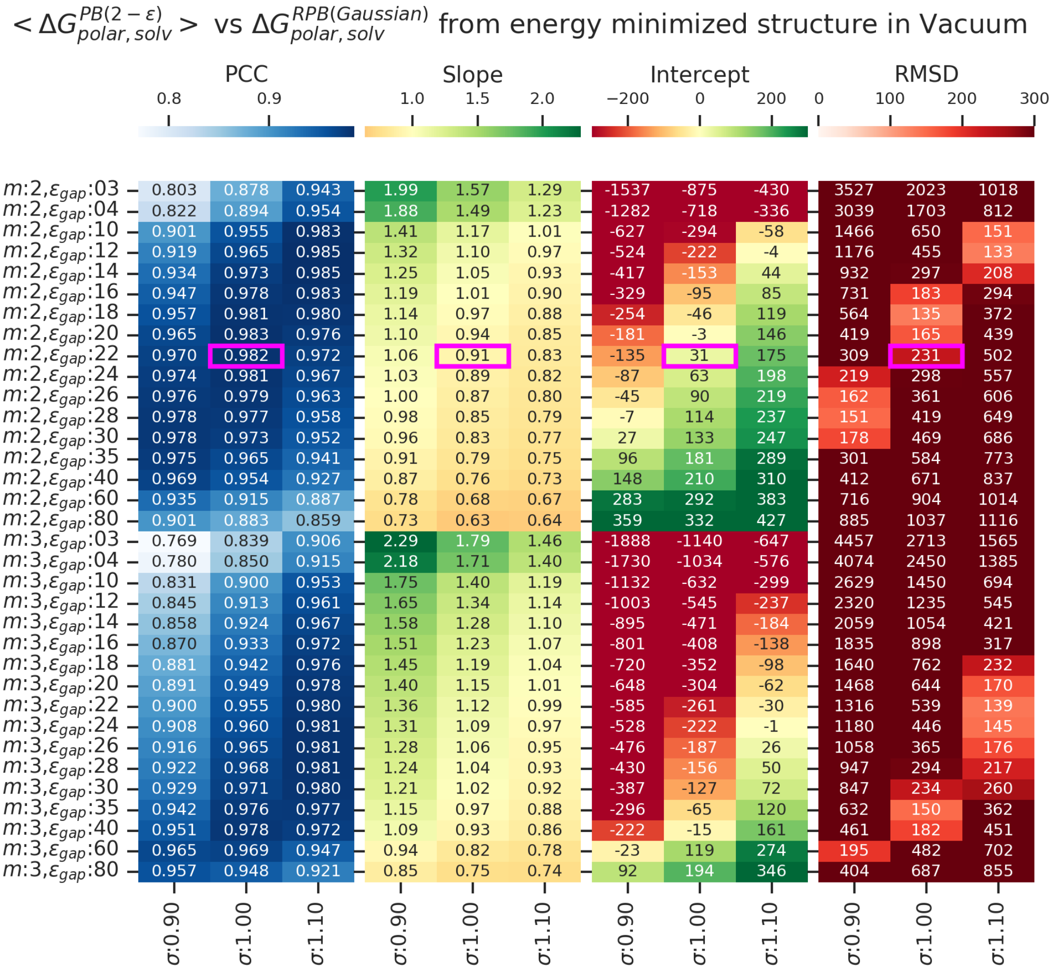

FIGURE S4 Summary of $\Delta G^{solv;polar}_{RPB}$ obtained from single energy minimized structures in vacuum using a super-Gaussian regularized PB method. The four performance indicators PCC, slope, intercept (in kcal/mol) and the RMSD (in kcal/mol) are shown as heatmap for a subset (showing only for better performing) of parameters explored. The optimal parameters we found are Gaussian exponent $m = 2$, limiting solute dielectric $\epsilon_{gap} = 22$, and variance of the super-Gaussian distribution $\sigma = 1.0$.
